# A rare case of myocardial bridging as a cause of complete heart block: A diagnostic challenge

**DOI:** 10.1016/j.radcr.2025.01.003

**Published:** 2025-01-18

**Authors:** Ibrahim Fathallah, Ayham Qatza, Ahmed Al-Talep, Reham Yousef, Rami Asef Hasn

**Affiliations:** a Faculty of Medicine, Al-Baath University, Homs, Syria; b Faculty of Medicine, Hama University, Hama, Syria; c Faculty of Pharmacy, Damascus University, Damascus, Syria; d Department of Cardiology, Albasel Hospital, Tartous University, Tartous, Syria; e Faculty of Medicine, Tishreen University, Latakia, Syria

**Keywords:** Complete heart block, Myocardial bridging, Catheterization, Case report

## Abstract

Complete heart block (CHB) is a disruption in electrical impulses to travel from atria to ventricles and can rarely be caused by myocardial bridging (MB), where cardiac tissue compresses a coronary artery during systole. The incidence of MB ranges from 0.5 % to 16 % in coronary angiography patients. This case report presents a 30-year-old female presented with dizziness, shortness of breath, and chest pain, diagnosed with third-degree AV block. Echocardiography revealed interventricular septal thickening and mild mitral regurgitation. Coronary angiography identified myocardial bridging in the mid LAD artery causing significant systolic stenosis. After ruling out reversible causes, a dual-chamber permanent pacemaker was implanted due to persistent heart block. The patient remained stable postprocedure, with decreasing cardiac biomarkers, and was discharged symptom-free with a follow-up appointment scheduled. MB can lead to serious cardiovascular events, including myocardial infarction and CHB. Clinicians must recognize the risks associated with MB and maintain a high suspicion for CHB to ensure timely management. Further studies are needed to clarify the CHB-MB relationship and improve patient outcomes.

## Introduction

The cardiac conduction system includes specialized fibers that originate electrical impulses and transport them across the heart's chambers [1]. May have an anatomical or physiological problem in the heart's conduction system, such as a complete heart block (CHB), which is a delay or disruption in the movement of an impulse from the atria to the ventricles [2]. (CHB) can be rarely caused by a myocardial bridging (MB). MB is the constructed tunnel's systolic pressure in the coronary artery by the cardiac tissue that covers it, which entirely vanishes during diastolic [3]. MBs are primarily located in the central region of the left frontal descending coronary artery [4]. In addition, diagonal and peripheral branches may be affected in 18 % and 40 % of situations, respectively. According to coronary angiography, the predicted incidence of their finding ranges from 0.5 % to 16 % [5]. This report presents a case of a 30-year-old female diagnosed with constrained myocardial bridging of the left anterior descending (LAD) artery, causing irreversible CHB. Following a successful dual-chamber permanent pacemaker procedure, the patient's condition stabilized.

### Case presentation

**Fig. 1:**
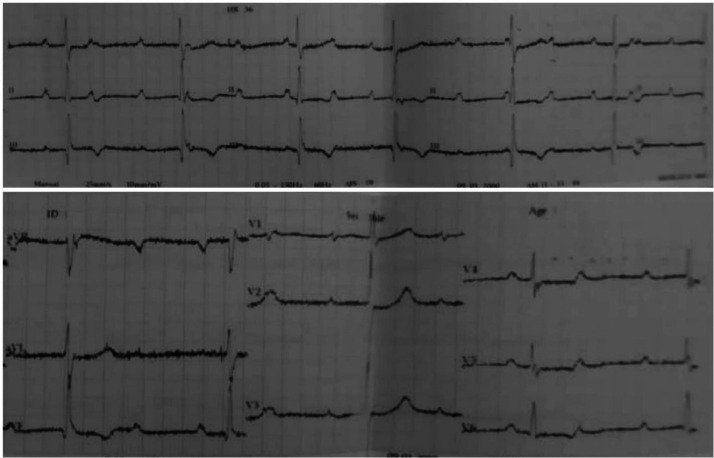
The 12-lead electrocardiogram (ECG) demonstrated a third-degree atrioventricular block "complete heart block".

**Fig. 2:**
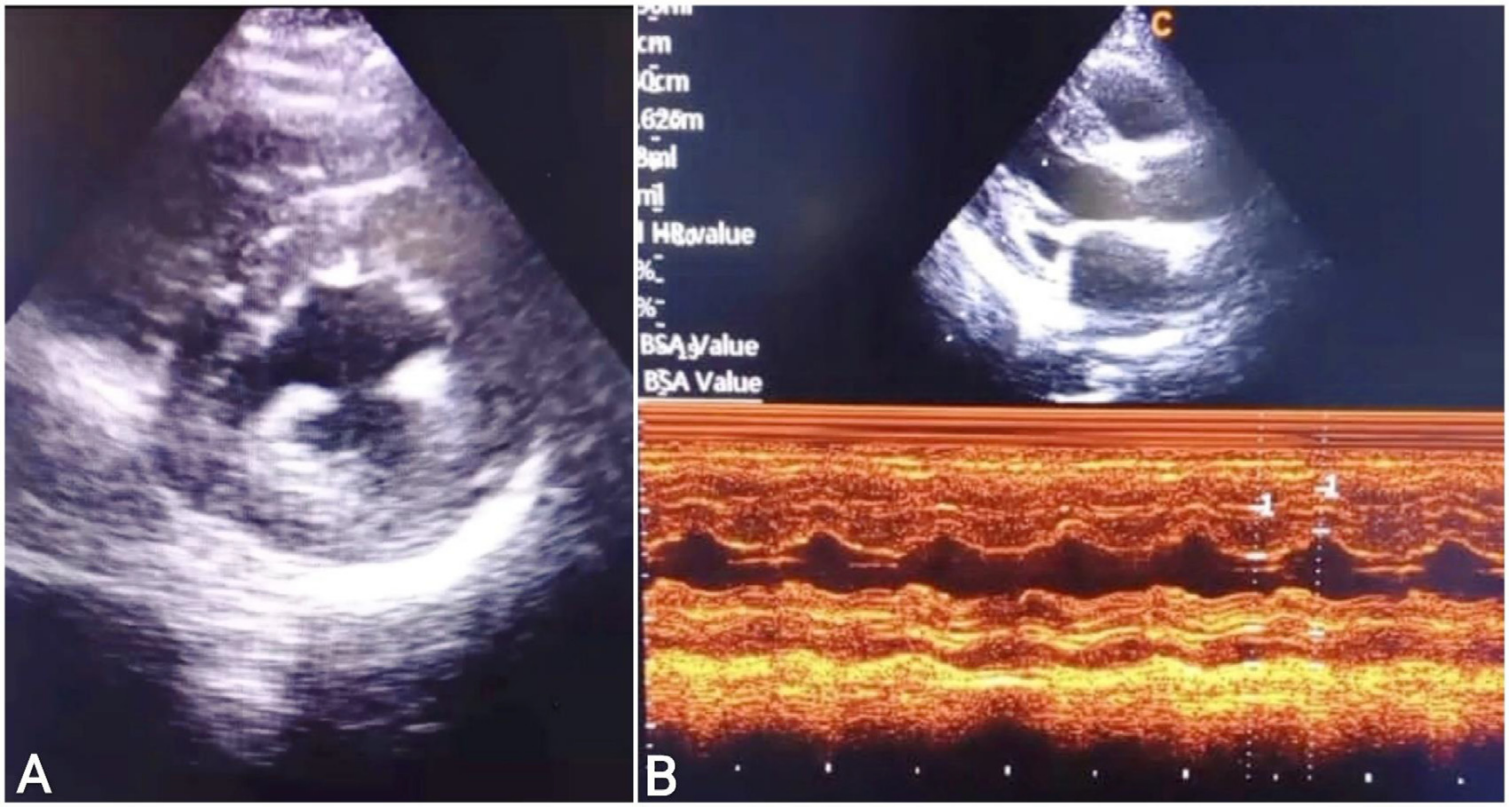
The two-dimensional transthoracic echocardiography: Parasternal short axis view (A) and parasternal long axis view (B) echocardiogram demonstrated left ventricular hypertrophy.

**Fig. 3:**
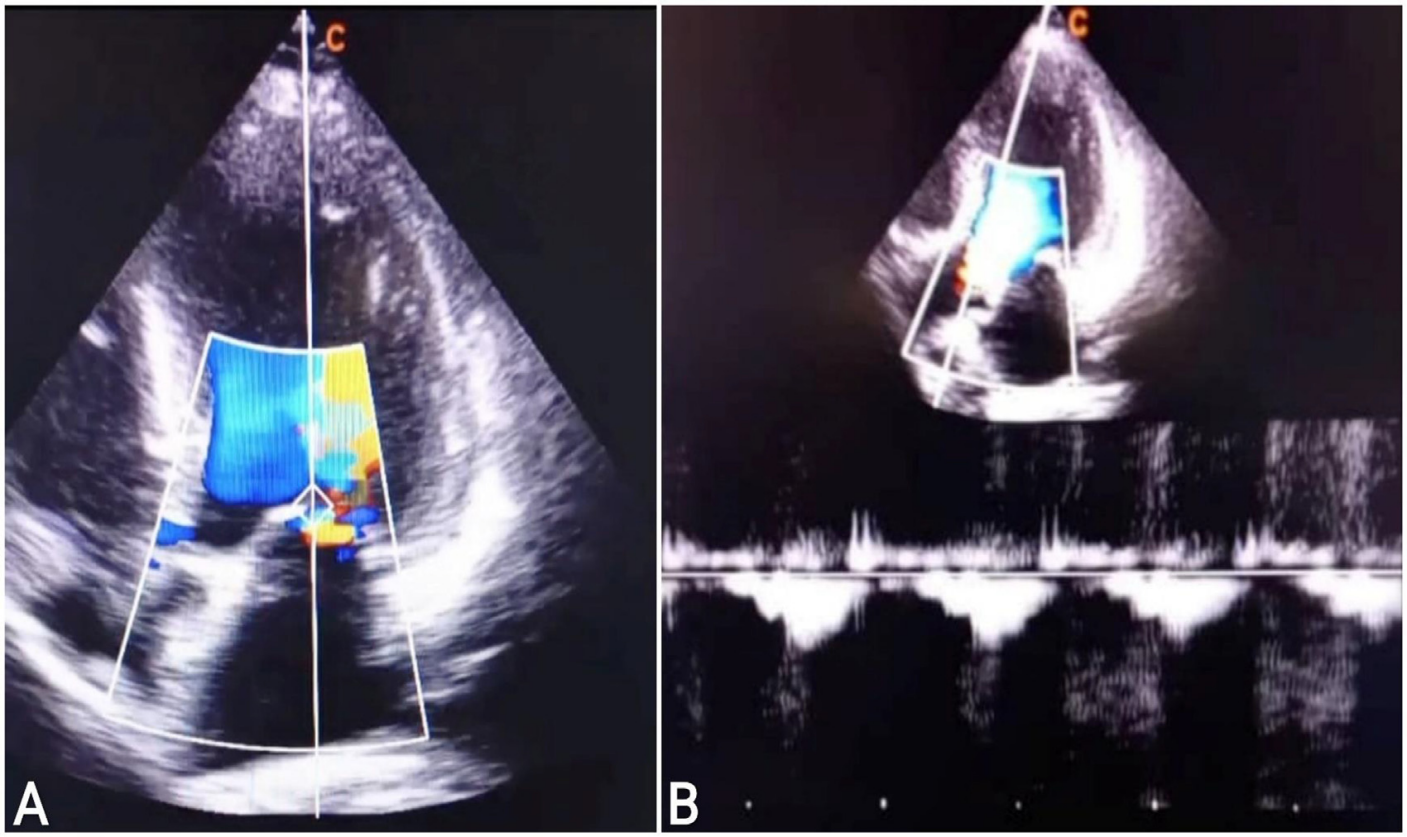
The two-dimensional transthoracic echocardiography. (A): The apical 4-chamber view echocardiogram displayed mild mitral regurgitation. (B): Continuous wave doppler of the normal aortic valve without stinosis or regurgitation.

**Fig. 4:**
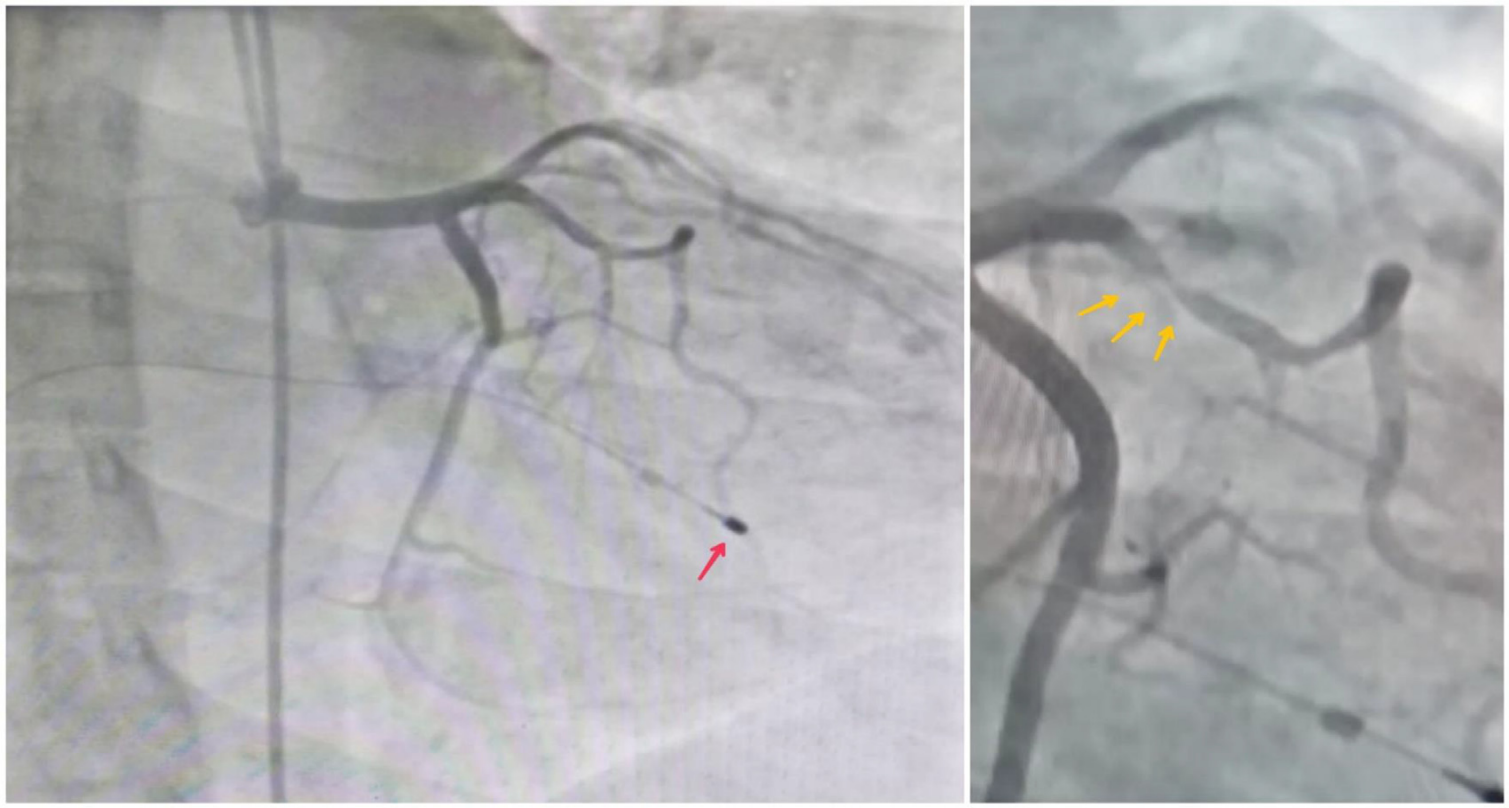
Invasive coronary angiography images: The left coronary angiogram revealed a myocardial bridge in the mid left anterior descending artery (yellow arrows), resulting in 90%-95% stenosis during systole (B), with normal caliber during diastole (A). In addition to a temporary pacemaker (red arrow).

A 30-year-old married female presented to the cardiac clinic with dizziness, shortness of breath, and 2 days of chest pain. She characterized the pain as pressure-like, severe in intensity, nonradiating, appearing at rest and worsening with exertion, and having no alleviating factors. No disease was reported in the patient's familial history. She was pregnant for the second time without any complications in the recent pregnancies and had previously undergone 1 caesarean section. She denied smoking, drinking alcohol, or drug abuse. Vital signs on admission were as follows: blood pressure, 90/60 millimeters of mercury (mm Hg); a slow heart rate, 35 beats per minute (beats/minute); tympanic temperature, 36.5 degrees Celsius (°C); and oxygen saturation, 93 % on room air. On physical examination, the patient was conscious and responsive with general weakness, pallor, clear-to-auscultation lungs, and systolic murmur heard during cardiac examination because of slight mitral regurgitation. Moreover, the neurologic and abdominal examinations were normal. All laboratory investigations were normal, except for an elevated troponin T level of 24 ng/mL. The 12-lead electrocardiogram (ECG) showed third-degree atrioventricular (AV) block"CHB" (Fig. 1). A two-dimensional transthoracic echocardiogram (2D-TTE) revealed an interventricular septal thickness (IVSS) of approximately 2 cm during systole (Fig. 2), mild mitral regurgitation, and a left ventricular ejection fraction (LVEF) of 65 % (Fig. 3). To address the CHB, a temporary pacemaker wire was inserted via the femoral vein into the right ventricle. Based upon these findings, the diagnosis was an acute myocardial infarction causing reversible complete heart block, which often improves with reperfusion. The patient underwent urgent percutaneous coronary intervention (PCI) in the cardiac catheterization laboratory to determine the cause. The left coronary angiogram revealed a MB in the mid LAD artery, resulting in 90%-95% stenosis during systole, with normal caliber during diastole (Fig. 4). Based on the angiographic findings, a coronary spasm provocation test was not conducted. Intravascular ultrasonography (IVUS), fractional flow reserve (FFR), and optical coherence tomography (OCT) were not conducted as they were unavailable in our setting. The patient was started on a loading dose of 300 mg aspirin, 300 mg clopidogrel, and 40 mg rosuvastatin.Following medication administration, reversible causes of CHB were ruled out. Despite 72 h of observation, there was no improvement in the heart block, leading to the implantation of a dual-chamber permanent pacemaker due to the persistent heart block and the patient's refusal for surgery. It is noteworthy that surgery does not guarantee the reversal of CHB or the return of sinus rhythm. The remainder of his ensuing hospitalization was uneventful, remaining angina-free and hemodynamically stable with decreasing cardiac biomarkers. He was discharged with a follow-up appointment scheduled in 1 week at the cardiology clinic. At the last review, he was in excellent condition without symptoms.

## Discussion

A third-degree heart block known as CHB happens when there is no AV conduction and no P waves linked to QRS complexes. Declared alternatively, the generated supraventricular impulses do not conduct to the ventricles. Rather, a junctional or ventricular escape rhythm maintains ventricular conduction, considering it exists. The atria and ventricles are entirely distinct from one another [6]. Patients with MB can present medically in one of 2 ways: by direct compression of the tunneled segment due to contraction of MB fibers or by acceleration and stimulation of atherosclerosis in the segment nearest to MB [7]. During systole, full or partial blockage of the descending anterior coronary artery can lead to significant MB. Patients with MB frequently suffer from tachycardia and other symptoms during workouts. It is considered that systolic compression was not the sole cause of ischemia because myocardial perfusion occurs mainly during diastole. Endothelial cell damage, chronic mechanical stress that predisposes the tunneled segment of a coronary artery to vasospasm, and early atherosclerosis on MB can all contribute to ischemia [8]. At rest, the patient may have chest squeezing [9]. Acute coronary syndromes, myocardial ischemia, myocardial spasm, exercise-induced dysrhythmias (such as ventricular tachycardia or AV block), transient ventricular dysfunction, syncope, or even sudden death are all possible signs of MB [10]. Previous research has shown that MB is more common in cardiac disorders related with left ventricular hypertrophy (LVH), such as aortic stenosis and hypertrophic cardiomyopathy (HCM) [11]. In particular, HCM patients have an increased incidence than the general population, with rates up to 30 % [12]. Myocardial ischemia appeared by persistently high troponin levels [13]. This case presents a rare instance of third-degree AV block "CHB" caused by MB, with limited reports available in the literature similar to it. According to a study, patients with MB can have minor myocardial dysfunction identified by three-dimensional speckle-tracking echocardiography predicated on the amplitudes of longitudinal, circumferential, and radial strains [14]. Echocardiography is advised for all patients with AV block in order to cancel out structural heart disease [15]. For treatment, antiplatelet therapy should be given consideration because patients with MB are more inclined to experience atherosclerosis. Beta-blockers are the primary type of conservative treatment for symptomatic patients because they reduce heart rate, increase diastolic coronary filling, and decrease myocardial contractility and coronary artery pressure. Nitroglycerin can be administered cautiously [16]. In addition, consider a temporary transvenous pacemaker for patients with unstable AV block who are not responding to medicinal treatment [17], and permanent pacemaker implantation needs to be explored in patients with high-grade or complete heart block [18]. Depending on present guidelines, dual-chamber pacing is better than single-chamber pacing [19]. Herein, a dual-chamber permanent pacemaker was implanted due to persistent heart block, as the patient declined surgical intervention.

## Conclusion

MB is an unexpected disease associated with a series of significant cardiovascular events, including myocardial infarction, arrhythmia, and sudden death. Therefore, it is crucial for clinicians to be aware of the potential risks associated with MB, and appropriate management should be implemented promptly. In addition, this paper aims to emphasise the importance of having a high suspicion of CHB in the patients diagnosed with MB because of its adverse and life-threatening consequences. Further studies and reports of similar cases can contribute to an enhanced comprehension of the CHB-MB relationship, thereby improving patient outcomes.

## Availability of data and materials

Data sharing not applicable to this article as no datasets were generated or analyzed during the current study.

## Ethics approval and consent to participate

Ethics clearance was not necessary since the University waives ethics approval for publication of case reports involving no patients' images, and the case report is not containing any personal information. The ethical approval is obligatory for research that involve human or animal experiments.

## CRediT authorship contribution statement

**Ibrahim Fathallah:** Writing—review and editing, Writing—original draft, Data curation.

**Ayham Qatza:** Writing—review and editing, Writing—original draft.

**Ahmed Al-Talep:** Writing—review and editing, Writing—original draft.

**Reham Yousef:** Writing—review and editing, Writing—original draft.

**Rami Asef Hasn:** Writing—review and editing, Supervisor.

**Ibrahim Fathallah:** Submitted the final manuscript.

All authors read and approved the final manuscript.

## Patient consent

Written informed consent was obtained from the patient for publication of this case report and any accompanying images. A copy of the written consent is available for review by the Editor-in-Chief of this journal on request.
